# Exploiting genotyping by sequencing to characterize the genomic structure of the American cranberry through high-density linkage mapping

**DOI:** 10.1186/s12864-016-2802-3

**Published:** 2016-06-13

**Authors:** Giovanny Covarrubias-Pazaran, Luis Diaz-Garcia, Brandon Schlautman, Joseph Deutsch, Walter Salazar, Miguel Hernandez-Ochoa, Edward Grygleski, Shawn Steffan, Massimo Iorizzo, James Polashock, Nicholi Vorsa, Juan Zalapa

**Affiliations:** Department of Horticulture, University of Wisconsin, Madison, Wisconsin USA; Instituto Nacional de Investigaciones Agrícolas, Forestales y Pecuarias, Campo Experimental Pabellón, Aguascalientes, Mexico; Department of Statistics, University of Wisconsin, Madison, Wisconsin USA; Valley Corporation, Tomah, Wisconsin USA; USDA-ARS, Vegetable Crops Research Unit, University of Wisconsin, Madison, Wisconsin USA; Department of Horticultural Sciences, Plants for Human Health Institute, North Carolina State University, Kannapolis, North Carolina USA; USDA-ARS, Genetic Improvement of Fruits and Vegetables Laboratory, Chatsworth, New Jersey USA; Blueberry and Cranberry Research and Extension Center, Rutgers University, Chatsworth, New Jersey USA

**Keywords:** Genotyping by sequencing, Imputation, *Vaccinium macrocarpon*, Pseudo-testcross, Linkage disequilibrium, Segregation distortion, Synteny

## Abstract

**Background:**

The application of genotyping by sequencing (GBS) approaches, combined with data imputation methodologies, is narrowing the genetic knowledge gap between major and understudied, minor crops. GBS is an excellent tool to characterize the genomic structure of recently domesticated (~200 years) and understudied species, such as cranberry (*Vaccinium macrocarpon* Ait.), by generating large numbers of markers for genomic studies such as genetic mapping.

**Results:**

We identified 10842 potentially mappable single nucleotide polymorphisms (SNPs) in a cranberry pseudo-testcross population wherein 5477 SNPs and 211 short sequence repeats (SSRs) were used to construct a high density linkage map in cranberry of which a total of 4849 markers were mapped. Recombination frequency, linkage disequilibrium (LD), and segregation distortion at the genomic level in the parental and integrated linkage maps were characterized for first time in cranberry. SSR markers, used as the backbone in the map, revealed high collinearity with previously published linkage maps. The 4849 point map consisted of twelve linkage groups spanning 1112 cM, which anchored 2381 nuclear scaffolds accounting for ~13 Mb of the estimated 470 Mb cranberry genome. Bin mapping identified 592 and 672 unique bins in the parentals and a total of 1676 unique marker positions in the integrated map. Synteny analyses comparing the order of anchored cranberry scaffolds to their homologous positions in kiwifruit, grape, and coffee genomes provided initial evidence of homology between cranberry and closely related species.

**Conclusions:**

GBS data was used to rapidly saturate the cranberry genome with markers in a pseudo-testcross population. Collinearity between the present saturated genetic map and previous cranberry SSR maps suggests that the SNP locations represent accurate marker order and chromosome structure of the cranberry genome. SNPs greatly improved current marker genome coverage, which allowed for genome-wide structure investigations such as segregation distortion, recombination, linkage disequilibrium, and synteny analyses. In the future, GBS can be used to accelerate cranberry molecular breeding through QTL mapping and genome-wide association studies (GWAS).

**Electronic supplementary material:**

The online version of this article (doi:10.1186/s12864-016-2802-3) contains supplementary material, which is available to authorized users.

## Background

The advent of next generation sequencing (NGS) technologies, coupled with reduced representation genome sequencing strategies, such as genotyping by sequencing (GBS), can generate vast quantities of single nucleotide polymorphism (SNP) markers in minor crop species lacking extensive genomic resources [1]. SNPs, the most common type of polymorphism in the genome, allow the construction of high-density linkage maps and concomitant identification of molecular markers tightly linked to complex traits of interest, known as quantitative trait loci (QTL). Since the introduction of linkage analysis by Sturtevant [2], researchers have improved and applied genetic mapping techniques in numerous commercially important species [3, 4]. Traditionally, low-throughput markers such as restriction fragment length polymorphisms (RFLPs) and simple sequence repeats (SSRs) were the molecular markers of choice for developing linkage maps of biparental populations and to anchor, order and orientate contigs, scaffolds, superscaffolds, and pseudo-chromosomes into physical maps [5–7]. Currently, SNPs are becoming more important for genetic, genomic, and molecular breeding research because they can be generated efficiently using NGS methods [1], and they are replacing RFLP and SSR markers for both major and minor crops.

Multiplexing techniques have been adapted for the GBS approaches to concurrently sequence multiple genotypes at many specific DNA sites across the genome [1, 8–11]. The multiplexing GBS pipeline developed by Elshire et al. [1] has been successfully used to produce large SNP data sets for several species (http://www.biotech.cornell.edu/) with or without reference genomes for the creation of high density linkage maps [12]. In addition, deeper sequencing and imputation methods are being developed to solve sequencing errors of NGS data, which cause difficulties during *de novo* SNP calling when reference genomes are unavailable [13, 14].

Besides allowing the construction of high density genetic maps, NGS technologies enable the implementation of QTL detection strategies with a higher density of markers, allowing for a more accurate detection of linked loci. In the past, most researchers performing QTL studies have operated under the assumption that the genome cannot be fully covered with markers, and therefore, have relied on a relatively small number of markers per linkage group to detect marker-trait associations using interval and composite interval QTL mapping [15]. In addition to providing a large number of markers that can be applied to traditional QTL methods, large SNP datasets allow researchers to characterize complex population structures, linkage disequilibrium (LD), and segregation distortion and to perform genome-wide association studies (GWAS) [16–18]. GBS currently provides a cost-efficient, high-throughput method with enough power to develop saturated linkage maps in biparental populations for QTL-mapping studies in almost any plant species. As a result, high density SNP linkage maps have been constructed for several minor crop and fruit crops species such as pear [19] and raspberry [20]. More recently, GWAS studies are being initiated and conducted based on SNPs in model fruit crops such as grape and apple [21, 22]. Both QTL and GWAS approaches could be especially useful in woody perennial fruit crop species where breeding and selection is impeded by the long generation interval, biennial bearing, adaptation to all seasons, etc., resulting in a long-term process requiring much field space, expensive and complex infrastructure, and intensive cultural management [22–24].

The *Vaccinium* genus, in the Ericaceae family, comprises more than 126 genera of perennial flowering plants and 4000 species commonly adapted to poor and acidic soils or epiphytic environments. Several *Vaccinium* species such as cranberries (*V. macrocarpon, V. oxycoccos*), blueberries (*V. corymbosum, V. darrowii, V. ashei, V. angustifolium*, etc.), and lingonberries (*V. vitis-idaea*) are specialty crops of economic importance [25–28]. Nevertheless, *Vaccinium* species have been understudied and their molecular and genomic characterization has been minimal until the recent advent of NGS technology [5, 29, 30]. Advances in cranberry genetics have been comparatively slow among fruit crops such as apple, peaches or blueberries, principally due to its recent domestication in the mid-1800s, the lack of private and publically funded research, and the slow selection progress inherent to woody perennial species [31–33]. In addition, cranberry breeding methods have relied solely on phenotypic selection with restricted experimental designs and limited genetic information or molecular genetic resources. Recently, NGS has been applied to generate a cranberry draft nuclear genome assembly and reference transcriptome [30], complete chloroplast [34] and mitochondrial [35] genomes, and moderate density linkage maps containing SSR, RFLP, and SCAR markers [36, 37]. The continued development of molecular tools such as high density linkage maps could increase cranberry selection efficiency and accuracy, especially for QTL introgressed from valuable wild germplasm resources [17, 25, 38]. Furthermore, NGS-based SNPs will enable GWAS and genomic selection in cranberry and other minor crop species using strategies currently being successfully employed in commodity crops such as maize, wheat, and soybeans [18] and model fruit crops such as apple and grape [21, 22].

The current study was initiated to generate a large SNP dataset using GBS in order to: 1) develop a saturated cranberry linkage map, 2) characterize genome-wide recombination, linkage disequilibrium, and segregation distortion, 3) anchor available cranberry genomic scaffolds and putative coding DNA sequences (CDS) [30] for candidate gene discovery, and 4) conduct an initial assessment of synteny between cranberry and other species. GBS was performed using multiplexed Illumina HiSeq sequencing based on Elshire et al. [1] and missing data was imputed using linear discriminant analysis (LDA) imputation methodology based on singular value decomposition [13, 14, 39] in a segregating biparental population (pseudo-testcross) consisting of 362 progeny derived from a cross between *[BGx(BLxNL)]95* and *GH1x35* (from now on referred as P_1_ and P_2_, respectively).

## Results

### Genotyping by sequencing

*Eco*T221 digested DNA from cranberry parental plants (repeated 8 times each) and progeny (*n* = 362) were sequenced yielding 3,000,842,566 total reads and 7,213,626 tags after merging. P_1_ accounted for 12,189,203 reads, whereas P_2_ accounted for 16,185,112 reads, each of the 362 siblings accounted for 8,348,193 reads on average. The samples were divided into four 96-well plates, and a linear model of the form y = Xβ + ε found significant differences between the number of reads per plate and per column, mainly due to variation in initial DNA concentrations and quality of the sample. A similar model was fitted to detect differences in missing data due to library preparation (plates) and samples (Additional file 1).

### SNP filtering, imputation, and segregation

After filtering to remove sequence tags with high levels of missing data (>20 %) and sequencing errors, 21,122 putative biallelic SNPs were detected in the cranberry mapping population. R scripts were used to further reduce the number of SNPs to 10,842 by excluding loci with a minor allele frequency (MAF) < 0.10 [13]. To maximize the amount of SNPs and genotypic data available for linkage mapping, median, principal components and linear discriminant imputation methods were tested to impute missing SNP values. Extensive simulations were performed for all methods to find the number of linear discriminants (LDs) and principle components (PCs) providing the lowest classification error. Linear discrimination analysis (LDA) imputation, which has been accepted and used as an accurate imputation method [13, 14, 39–41], yielded the lowest classification error between the methods tested in the SNP dataset by using 80 LDs (~9.7 % classification error). Following imputation, highly distorted loci (*p*-value < 0.001) resulting from sequencing errors and/or other unknown biological mechanisms were discarded prior to map construction and only loci with mild levels of distortion *p*-values > 0.001 were included. Therefore, 5,477 segregating SNPs were selected for further analysis in addition to 211 SSR markers previously reported by Schlautman et al. [37]. Of these markers, 1977 markers were heterozygous in the parental configuration ABxAA (P_1_), 2370 markers in the configuration AAxAB (P_2_), 1273 were heterozygous in the configuration ABxAB (both parents) and 68 markers of configuration ABXCD (4 alleles). Loci were further separated into 2 sets of uniparental configurations (ABxAA and AAxAB) to create parental bin maps.

### Recombination estimation and linkage mapping

**Fig. 1:**
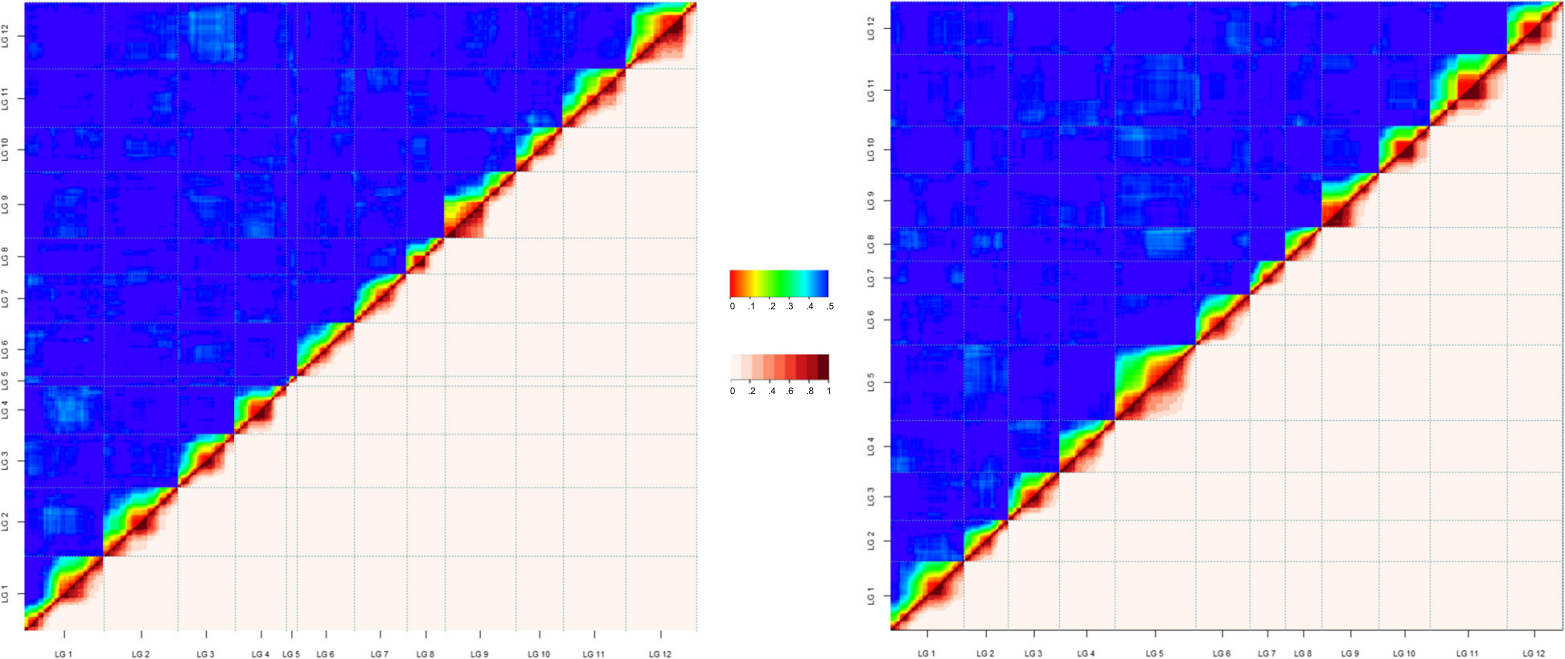
Cranberry parental linkage map genome-wide recombination frequency and linkage disequilibrium patterns. Ordered markers in *[BGx(BLxNL)]95* (P_1_; A) and *GH1x35* (P_2_; B) parental bin maps for the 12 linkage groups containing 1728 and 2021 molecular markers (type ABxAA and AAxAB), comprising 592 and 671 bins respectively, were used to show the computed parsimonious order of the map. The upper triangular region of the matrix (in red palette) shows the linkage disequilibrium (calculated using the r^2^ metric) whereas the lower triangular shows the recombination between markers (in rainbow palette)

Using the parental configurations, parental bin maps were generated using the minimum spanning tree (MST) algorithm implemented in ASMap [42], and final distances were obtained providing those fixed orders to JoinMap using the regression procedure [43]. Linkage groups (LGs) were determined with a LOD threshold >10, the Kosambi mapping function was used to calculate genetic distances between loci. The ASMap package in R [42] was used to obtain the order of loci and recombination matrices for the parental maps were calculated for graphical assessment of the MST ordering algorithm (Fig. 1), additional markers were discarded due to high number of genotyping errors or high number of double recombination events.

The final parental bin maps for P_1_ and P_2_ contained 1728 and 2021 uniparental markers corresponding to 592 and 671 bins, respectively (Additional file 2: Figure S1; Additional file 3: Tables S1 and S2; Table 1). The P_1_ bin map spanned a total of 1321 cM with a maximum and minimum LG length of 138.2 (LG1) and 74 cM (LG5), an average LG length of 110.1 cM, and an average marker interval of 2.5 cM. The P_2_ bin map spanned a total of 1137 cM, with a maximum and minimum LG length of 123 cM (LG1) and 78 cM (LG8), an average length of 94.7 cM, and an average marker interval of 1.73 cM (Table 1). The average, minimum and maximum number of recombination events per LG for the P_1_ bin map were μ = 1.1, 0.7 (LG5) and 1.4 (LG1), and for the P_2_ bin map were μ = 1, 0.8 (LG8) and 1.2 (LG1), respectively (Table 2).

**Table 1:** Features of the cranberry integrated (I) and parental bin linkage maps (P_1_ and P_2_)

LG	Length (cM)			Tot.No.Markers			No.SSR			No.SNP			#Bins		Avg.Gap (cM)	
	I	P_1_	P_2_	I	P_1_	P_2_	I	P_1_	P_2_	I	P_1_	P_2_	P_1_	P_2_	P_1_	P_2_
1	107.1	138.2	123.0	542	198	220	14	7	10	528	191	210	73	77	1.9	1.6
2	95.8	113.8	95.2	413	192	134	16	10	7	397	182	127	60	49	1.9	1.9
3	100.2	120.8	90.6	357	142	154	15	7	14	342	135	140	50	56	2.4	1.6
4	88.5	115.6	91.2	354	138	172	22	16	12	332	122	160	55	61	2.1	1.5
5	103.8	74.0	96.1	301	29	244	16	4	15	285	25	229	13	69	5.7	1.4
6	97.9	131.8	91.4	421	142	157	12	10	6	409	132	151	53	59	2.5	1.5
7	95.2	116.0	88.6	335	136	110	18	11	10	317	125	100	54	40	2.1	2.2
8	96.7	98.0	78.0	302	104	111	19	13	8	283	91	103	33	46	3.0	1.7
9	78.3	112.8	99.1	473	180	167	19	13	4	454	167	163	48	57	2.3	1.7
10	78.7	85.3	81.4	372	122	152	12	8	4	360	114	148	47	38	1.8	2.1
11	86.0	95.5	103.1	526	162	229	18	9	9	508	153	220	54	66	1.8	1.6
12	83.9	119.3	99.0	453	183	171	20	15	15	433	168	156	52	53	2.3	1.9
Total^a^	1112.1	1321.1	1136.7	4849	1728	2021	201	123	114	4648	1605	1907	592	671	2.5	1.7

Summary of total linkage group lengths, total number of markers, number of SSRs, SNPs, number of bins, and average gap in cM

^a^Summed across LGs = Length (cM), Tot.No.Markers, No.SSR, No.SNP, #Bins,; Averaged across LGs = Avg.Gap (cM)

**Table 2:** Genome-wide features of the cranberry integrated (I) and parental bin maps (P_1_ and P_2_)

LG	No.Dist.Mark			%Dist.Mark			#Rec.Event		LD.decay		No.genes			No.scaff			Mb		
	I	P_1_	P_2_	I	P_1_	P_2_	P_1_	P_2_	P_1_	P_2_	I	P_1_	P_2_	I	P_1_	P_2_	I	P_1_	P_2_
1	45	5	30	8.3	2.5	13.6	1.4	1.2	31.7	32.1	136	56	76	257	113	131	1.4	0.5	0.7
2	20	9	7	4.8	4.7	5.2	1.1	0.9	31.9	31.0	132	68	58	205	111	85	1.1	0.6	0.4
3	7	3	4	2.0	2.1	2.6	1.2	0.9	33.1	32.4	96	40	45	183	81	85	0.9	0.4	0.4
4	19	3	9	5.4	2.2	5.2	1.1	0.9	35.8	32.5	106	36	62	195	89	107	1.0	0.4	0.6
5	13	2	10	4.3	6.9	4.1	0.7	1.0	23.0	31.3	97	11	65	142	17	128	0.8	0.1	0.6
6	83	10	60	19.7	7.0	38.2	1.3	0.9	35.0	31.2	107	40	49	200	84	84	1.1	0.5	0.4
7	65	11	36	19.4	8.1	32.7	1.2	0.9	32.0	31.0	88	64	29	162	85	67	0.8	0.5	0.3
8	22	4	3	7.3	3.8	2.7	1.0	0.8	28.9	31.6	75	32	29	152	60	67	0.8	0.3	0.3
9	60	8	46	12.7	4.4	27.5	1.1	1.0	32.1	31.6	108	57	45	226	102	89	1.1	0.5	0.4
10	36	7	25	9.7	5.7	16.4	0.8	0.8	29.8	31.0	98	43	56	186	70	84	0.9	0.4	0.5
11	86	8	57	16.3	4.9	24.9	0.9	1.0	32.5	32.7	131	51	70	258	98	129	1.3	0.5	0.6
12	67	10	29	14.8	5.5	17.0	1.2	1.0	32.0	33.2	111	59	66	215	109	98	1.1	0.5	0.6
Total^a^	523	80	316	10.4	4.8	15.9	1.1	0.9	31.5	31.8	1285	557	650	2381	1019	1154	12.3	5.1	5.9

Summary of number of distorted markers, percent of distorted markers, number of recombination events, linkage disequilibrium (considered at *r* ^2^ = 0.2), and number of genes, scaffolds and Mb anchored

^a^Summed across LGs = No.Dist.Mark, No.genes, No.scaff, Mb; Averaged across LGs = %Dist.Mark, #Rec.Event, and LD.decay

### SSR homology and map comparisons

**Fig. 2:**
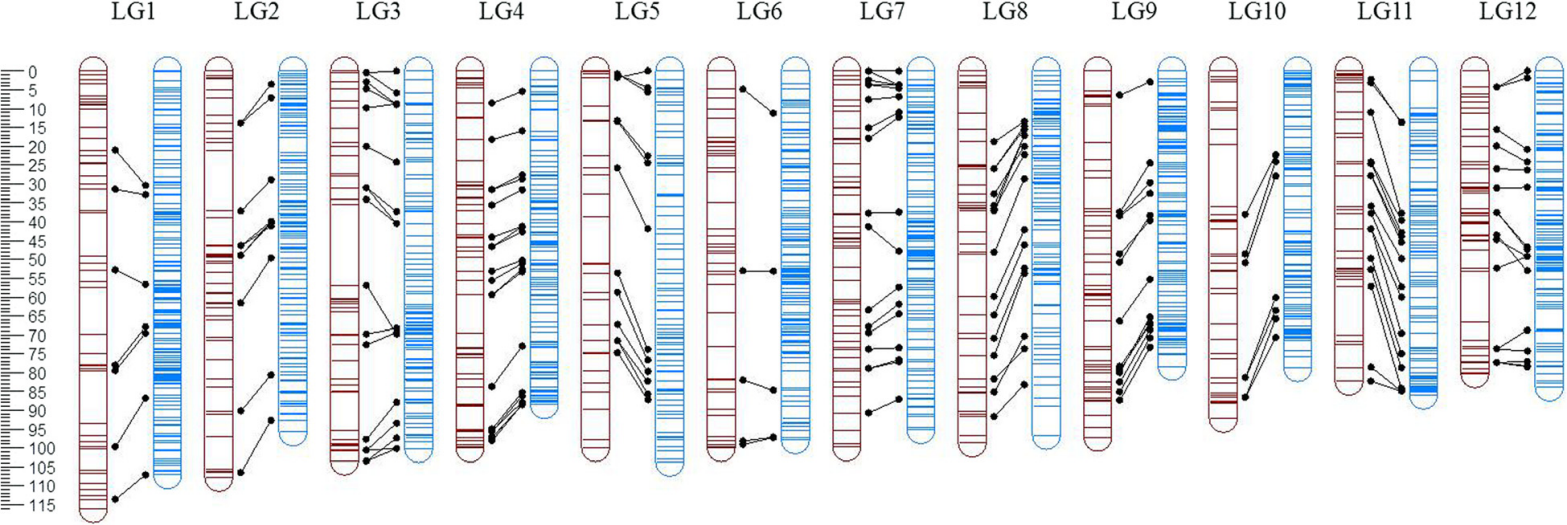
Cranberry SSR and SNP map homology. Homology between the SSR map (red LGs) developed by Schlautman et al. [44] and the SNP-SSR map (black) depicted by black dotted lines showing the accuracy of locus ordering using the MST and ML algorithms comprising 4849 markers

In order to construct an integrated map, a dataset containing uniparental and double heterozygote markers (ABxAA + ABxAB and AAxAB + ABxAB) was used and parental maps were constructed forcing the order found in the parental bin maps. The position of double heterozygote markers did not change across parental maps (Additional file 4: Figure S2). Finally, parental maps including double heterozygote markers were merged in an integrated map using JoinMap 4.1® (Additional file 3: Table S3). A total of 201 polymorphic previously mapped SSRs with normal segregation were positioned in the integrated map and 123 and 114 in each of the parental maps (Table 1). Comparison of SSR marker order in the Schlautman et al. [44] and the SNP-SSR integrated linkage map revealed consistent collinearity and validated the SNP positions and LG structure of the current high density cranberry linkage map (Fig. 2). The total integrated map spanned 1112 cM in length and contained 4849 markers with 1676 unique marker positions; the largest LG spanning over 107.1 cM (LG1) and the shortest spanning 78.3 cM (LG9) (Fig. 2; Table 1).

### Genome-wide segregation distortion and Linkage disequilibrium

**Fig. 3:**
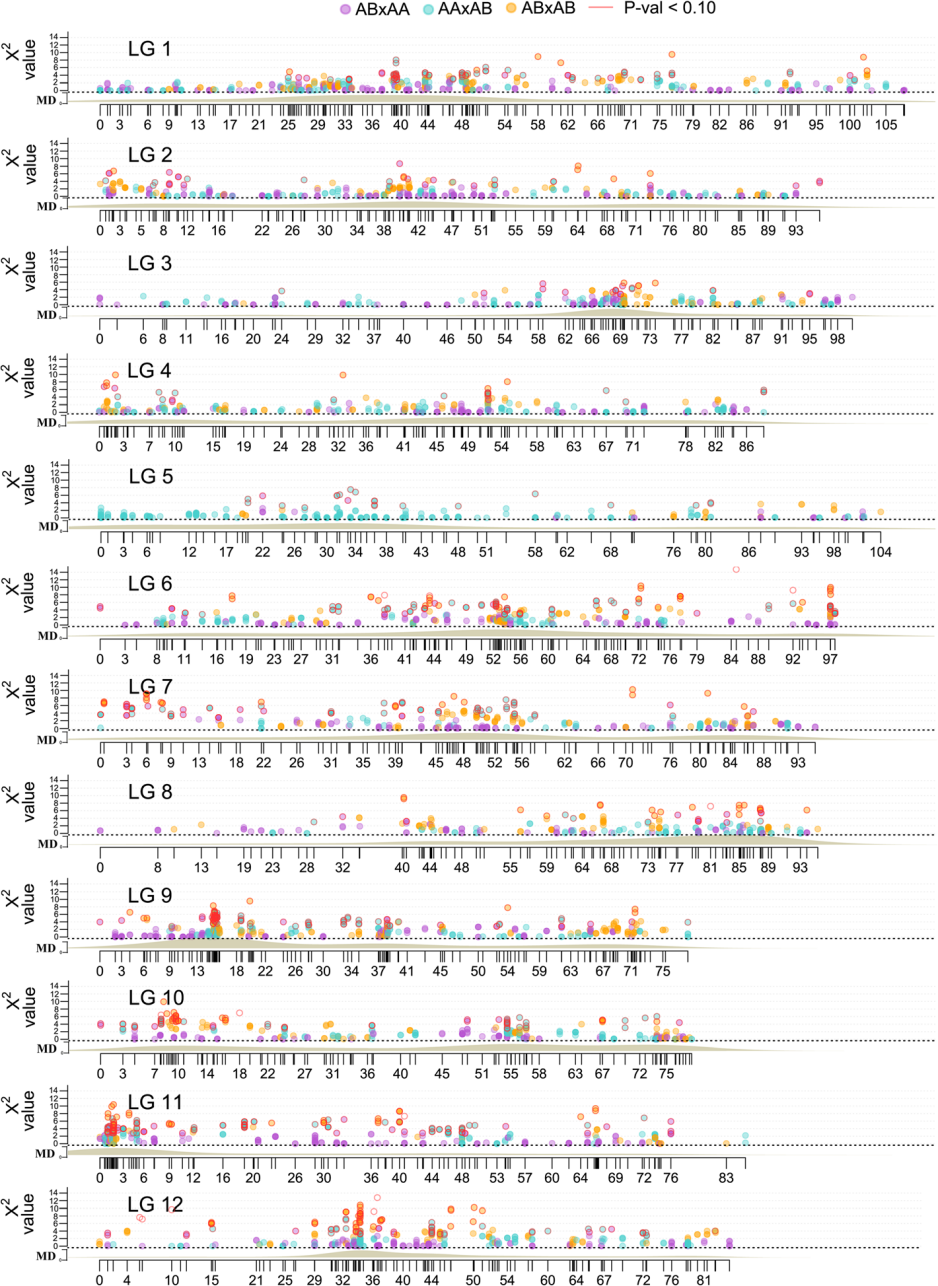
Cranberry linkage map marker density/depth and genome-wide patterns of segregation distortion. Segregation distortion is plotted as a function of Chi-squared value (y axis) for monogenic marker segregation ratios against marker position on each of the twelve LGs of the integrated map. Uniparental markers for P_1_ (female is heterozygote) are indicated with pink, uniparental markers for P_2_ (male is heterozygote) are displayed in light blue, whereas biparental markers (both parents heterozygotes) are shown in gold dots. Dots with red halo indicate markers with Chi-squared values significant at *p*-values ≤ 0.10. Genetic distances are displayed in cM on the x axis. MD label on the y axis refers to marker density across the linkage group

The overall number of distorted markers is presented in Table 2. Of the markers positioned in the integrated map, 10.4 % displayed some degree of segregation distortion (*p*-value < 0.10) according to *χ*^2^ tests with 1, 2 and 3° of freedom for the backcross (AAxAB, ABxAA), F_2_ (ABxAB) and F_1_ (ABxCD) type markers, respectively (Fig. 3; Additional file 3: Table S4). The distortion favored the homozygote configuration for the P_1_ (86 % of ABxAA distorted markers had more genotypes AA) and no specific allelic configuration was favored in P_2_ (58:42 % for AA:AB genotypes), but heterozygote configurations were more common in P_2_ (AAxAB) than in P_1_. To assess linkage disequilibrium in cranberry, only markers positioned in the parental bin maps and segregating in the uniparental AAxAB and ABxAA fashion with a unique segregation pattern were analyzed. Marker genotypes were transformed to 0:1 and 1:0 format, and were sorted by map position. LD across the 12 LGs appeared to be consistent in both the P_1_ and P_2_ parental backgrounds, and the observed patterns of LD among and between loci corroborated the marker order computed by ASMap and JoinMap (Fig. 1). In addition, the average, minimum, and maximum LD decay across the cranberry genome (Table 2) were computed for both parental bin maps (Additional file 5: Figure S3), which considering a decay at *r*^2^ = 0.2 were 31.5, 23.0 (LG5) and 35.8 (LG4) cM, respectively for P_1_ and 31.8, 31 (LGs 2,7,10) and 33.2 (LG12) cM for P_2_. The estimated equivalence of Kb per cM was ~422 Kb/cM, which indicates that LD in this biparental cranberry population can extend up to 13.39 Mb.

### Scaffold anchoring

The 4849 markers positioned in the high density linkage map comprised 2381 scaffolds from the cranberry nuclear assembly, representing ~13 Mb (12,318,679 bp) or 2.76 % of the total expected genome length (470 Mb) [30]. Approximately, 1285 previously identified and annotated predicted coding DNA sequences (CDS) were contained within the 2381 scaffolds anchored [30] (Table 2, Additional file 3: Table S5). Pseudo-molecules representing the 12 LGs were created by arranging the 2381 scaffolds according to their locus position in the integrated map in an attempt to anchor portions of the cranberry nuclear genome (Table 2). LG1 anchored the largest number of genomic sequence data (1,365,710 bp), while LG5 anchored the lowest number of nucleotides (768,990 bp). Similarly, LG1 anchored the largest number of CDS (136), while LG8 anchored the lowest number of CDS (75). Scaffolds anchored were not oriented.

### Initial synteny analysis with other fruit crop genomes

**Fig. 4:**
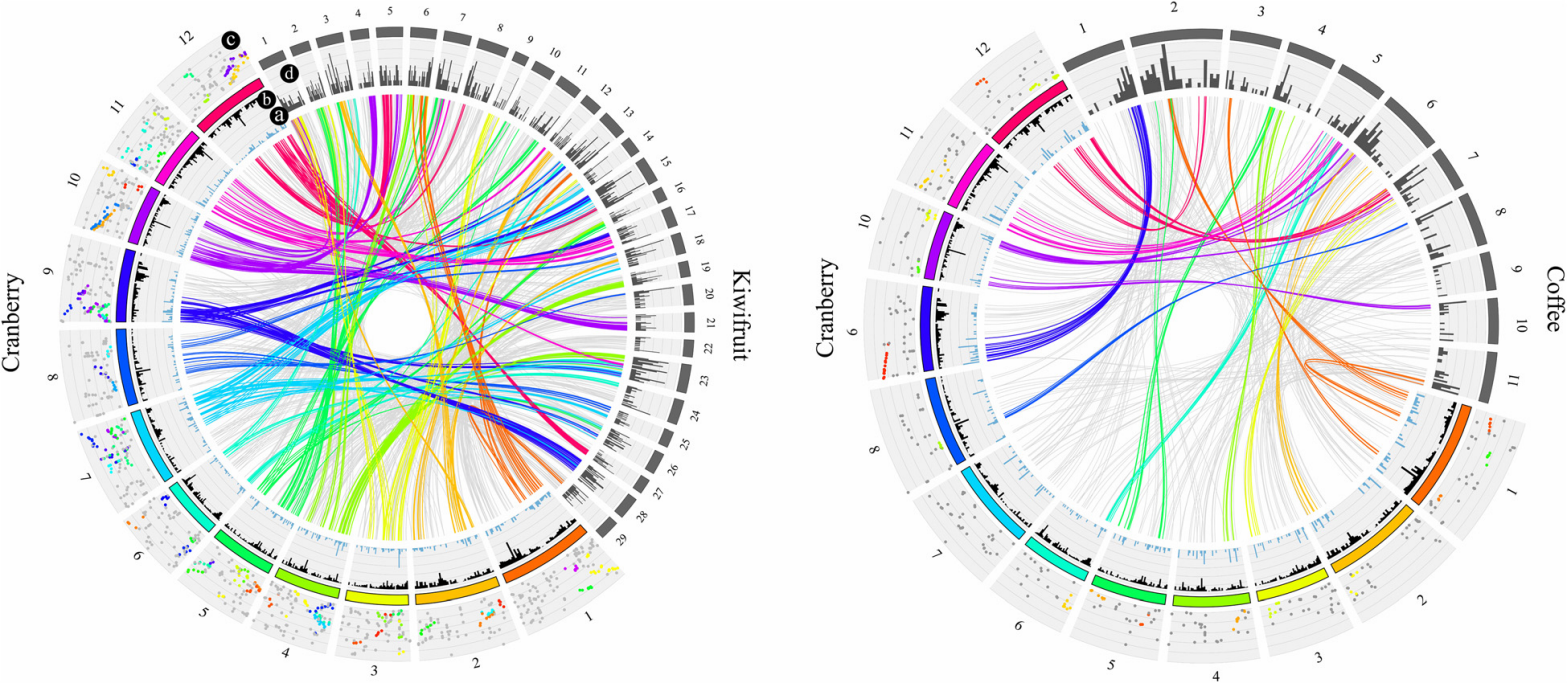
Synteny analysis. Synteny blocks between cranberry and kiwifruit (left panel) and cranberry and grape (right panel). The 12 cranberry chromosomes are represented in different colors whereas chromosomes for the other species have dark gray color. The distribution of significant BLAST hits between cranberry (ring a, blue histogram) and the other species (ring d, gray histogram) are shown. Ring b (black histogram) shows the SNP marker density in each cranberry LG. In order to visually explore syntenic blocks, the location of each homologous sequence between cranberry (x-axis) and the reference species (y-axis) was plotted in ring c. In this plot, different dot colors were used to differentiate chromosomes in the reference species; those hits that potentially could be syntenic blocks (see Methods), show non-gray color. For example, a section of the LG 10 in cranberry (purple lines) has homology to large portions of chromosomes 4 and 21 in kiwifruit (see blue and orange dots in ring c)

Local BLAST of 1113 CDS anchored in the cranberry genome identified 1,290, 615, and 421 homologous sequences in the kiwifruit, grape, and coffee genomes, respectively (Fig. 4; Additional file 6: Figure S4). By comparing the positions of the CDS anchored in the cranberry genome against their physical location in the genomes of kiwifruit, grape, and coffee, several syntenic regions were identified (Fig. 4; Additional file 3: Table S6-S8). For example, a major syntenic region covering almost one half of the LG9 in cranberry and the whole chromosome 28 in kiwifruit was identified (Fig. 4). In addition, the same region in LG9 in cranberry was syntenic with a portion of chromosome 1 of coffee. These and other microsyntenic regions identified among cranberry LGs with kiwifruit and coffee chromosomes resemble the close phylogenetic relationship among these species, specially between cranberry and kiwifruit which are both members of the Ericales (Fig. 4). Grape is another species evolutionarily related to cranberry for which a robust genome assembly is available. Several additional microsyntenic regions between the cranberry and grape were also identified (Additional file 6: Figure S4). Several microsyntenic blocks contained the same CDS in all four species (not shown) suggesting conservation of certain genomic regions among multiple distantly related taxa. Local BLAST of 1113 CDS anchored in the cranberry genome and the available blueberry (*V. corymbosum*; 2x = 2n = 24) draft genome assembly [29] containing 13,757 scaffolds revealed 2031 blueberry scaffolds potentially homologous. However, no microsyntenic regions could be identified due to the low level of assembly of the blueberry scaffolds and the lack of well-defined chromosomes (Additional file 3: Table S9). However, some blueberry scaffolds could be anchored into 12 pseudo-chromosomes under the assumption that few genomic rearrangements exist between blueberry and cranberry, which are both members of the *Vaccinium* genus.

## Discussion

NGS techniques allow the discovery of thousands of polymorphic markers useful for the construction of high density linkage maps in an understudied crops [5, 45–47]. We applied GBS approaches [1] to allow the massive development of SNPs in cranberry similar to major crops such as maize, wheat, and soybeans [18] and other model fruit crops such as apple and grape [21, 22]. After optimization with several enzymes, we selected EcoT221 to perform GBS, which yielded a conservative number of SNP with high coverage during marker calling (average of 46.5 reads per individual per marker). Additionally, we tested different imputation methods to diminish the problem of missing data (median, principal components and linear discriminant analysis). In our study, linear discriminant imputation possessed the lowest classification error using the first 80 LDs (~9.65 %) compared with median imputation (~22 %) and principal components imputation (14 %) [13, 14, 39, 41]. We used the pseudo-testcross strategy which has been used to overcome outcross difficulties in perennial crop species and provide information of the parental backgrounds [20]. To solve the marker ordering challenge during map construction [48, 49], we found it especially useful to use the minimum spanning tree (MST) algorithm implemented in ASMap once identical markers were removed to create bin maps and identified genotyping errors reducing the complexity of data [42, 50] and the maximum likelihood algorithm implemented in JoinMap v4.1 for outcrosses to fit maps based on orders found by the MST algorithm using all data [49]. Despite some challenges due to the nature of cranberry, GBS technologies enabled us to build an integrated map with 4849 markers with 1676 unique marker positions comprising 592 and 671 parental bins. This map is the densest linkage map ever developed within the *Vaccinium* genera and the entire Ericaceae family. Until recently, the development of a fully saturated cranberry map was an inconceivable achievement, and yet the current map was constructed at a fraction of the cost in time and economic resources than using traditional development methods based on AFLP, EST, or SSR markers [36, 37, 44, 51, 52].

### Comparison of the current genetic map with previous cranberry maps

We genotyped a population of 362 individuals derived from the cross among two highly heterozygous cranberry individuals (F_1_ cross) *[BGx(BLxNL)]95* x *GH1x35*. The integrated linkage map was constructed using a total of 4648 SNP and 201 SSR biparental and uniparental markers (Table 1). The twelve cranberry linkage groups constitute the expected chromosome number *n* = *x* = 12 [53–55] according to Schlautman et al. [44] and Georgi et al. [36] maps. The linkage map spanned 1112 cM, 5 % shorter and 26 % longer than the previous cranberry maps spanning 1177 cM and 880 cM, respectively [36, 44]. The improvement and length increase in current cranberry linkage map developed here is due to the addition of a large number of markers (n_SNP-SSR_ = 4849), which permitted an increased coverage of the genome, particularly in telomeric regions [56, 57] (Fig. 2). In fact, the addition of GBS-based SNPs resulted in a nearly 10-fold increase in marker coverage of the cranberry genome from the 541 previously mapped markers [44] (Fig. 2). Additionally, the current mapping effort used a much larger population than either of the two previous mapping efforts with 362 genotypes used versus 221 [44] and 182 [36] mapping individuals, which resulted in a greater accuracy on the number of recombination events detected (bins) [58]. Previously, decreased rates of recombination have been observed in the telomeric regions of some plant species, which may explain the lower density of markers in the SNP map found in some LGs such as LG8 and LG11 [20, 59, 60]. Thus, in addition to inherent differences between genetic backgrounds, the increased length of LGs observed in the current SNP-SSR map and the Schlautman et al. [44] map compared to the first linkage map [36] is likely due to the increment of power to detect recombination events by using a larger population coupled with higher marker density [61].

The use of SSRs in the current map in addition to the SNPs allowed us to detect almost near perfect collinearity with the Schlautman et al. [44] SSR map. In total 201 out of 211 SSRs were included in the integrated map, 13 which have not been previously reported (Additional file 3: Table S5, Table 1). Based on a comparison of homologous SSR markers among the current map and the Schlautman et al. [44] map, a few markers were inverted or positioned in slightly different locations (Fig. 2). Some studies have found that local inversions and minor discrepancies in marker positions are not uncommon during map integration or consesus map development [62–64]. Distal end rearrangements of closely linked markers have been reported in many species [20, 65]. Additionally, marker collinearity or synteny inconsistencies could reflect the true genomic structure between mapping populations. In fact, such genomic rearragments may actually be more common than expected and could represent regions of evolutionary plasticity involving selection and random drift [66, 67]. Interestingly, we found LGs with blocks of inverted markers consistent with balanced rearrangements consistent with double-stranded breaks between parental maps (Fig. 2; Additional file 4: Figure S2) [68, 69]. For example, LG8, LG9 and LG12 in our study showed some inverted markers compared with previous map [44] while the absence of a large portion of the maternal LG5 could be due to a previously reported cyclical translocation in cranberry [54, 55, 67] (Additional file 4: Figure S2).

### Comparison of current genetic map with a previous blueberry map

Blueberry (*Vaccinium corymbosum*; 600 Mb) is the closest relative of cranberry (470 Mb) and has the same basic chromosome number (x = n = 12) and similar expected genome size [29]. Current genetic mapping efforts in blueberry have yielded an interspecific diploid blueberry map (*V. darrowii* x *V. corymbosum*) F_1_ x *V. corymbosum* consisting of 265 markers, mainly SSR [51], spanning 1740 cM across 12 linkage groups. The blueberry map covered 89.9 % of the blueberry genome and reconstructed its expected 12 chromosomes. Similarly, we recovered the 12 cranberry linkage groups in 1112 cM in cranberry with an estimated coverage of 99.5 % according to method 4 of Chakravarti et al. [70] (Additional file 3: Table S10). Due to the lack of markers in common among blueberry and cranberry maps, an assessment of linkage group synteny and collinearity was not possible. Since all 43 *Vaccinium* species are closely related and have a basic chromosome number of 12, they can potentially be used for interspecific breeding purposes. For example, artificial interspecific hybrids have been reported among *V. macrocarpon × V. oxycoccos and V. macrocarpon × V. vitis-idaea, V. myrtillus × V. vitis-idaea, V. angustifolium × V. corymbosum × V. darrowii, V. darrowii x (V. macrocarpon x V. oxycoccos)* [71] (Covarrubias-Pazaran, unpublished results). Future comparative genetic mapping efforts should focus on mapping and synteny comparisons among multiple species across the genus to characterize the genomic features of closely related species and provide a better understanding of the evolutionary history and breeding potential of *Vaccinium* species.

### Cranberry saturated map in comparison with previous maps

The cranberry linkage map described herein was comparable to several high density maps developed recently with respect to the number of SNP mapped, overall marker density and gap lengths [19, 20, 57, 62, 72–74]. Interestingly, however, in some cases, high-density GBS genetic maps such as the raspberry map have found extremely different numbers of SNP per LG and numbers of SNPs assigned to each parental map [20]. A similar phenomena was found in rubber tree, where the genetic maps from two F_1_ progenies presented very different numbers of SNP markers assigned to each LG [62]. In our study, we have found fairly consistent numbers of markers per LG in both parental and integrated maps except for LG5 from the P_1_ parental map, which contained only 50 unique markers. The lack of markers of the ABxAA configuration might represent a signature of a previously reported cranberry cyclical translocation [54, 55]. A decreased ability to detect recombinant gametes for LG5 would be expected due the translocation given lower recombination and lack of chromosome pairing in the region, which would translate into dramatically reduced number of ABxAA type of markers in LG5 [66]. Although no cytological or sequence evidence is available to support our hypothesis, the genomic scaffold sequences containing markers integrated in our linkage maps open the opportunity to design probes for fluorescent in situ hybridization (FISH) experiments targeting the chromosome regions flanking this possible translocation.

### Recombination

**Fig. 5:**
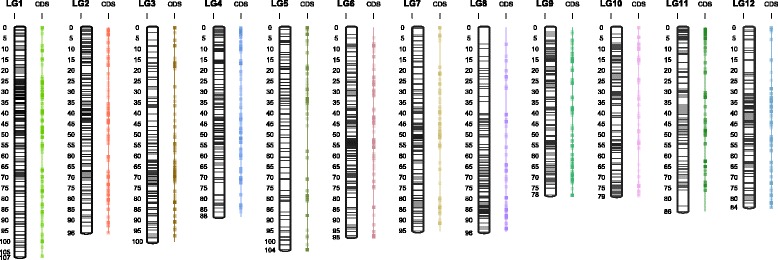
Cranberry SNP-SSR integrated linkage map with putative genes. Genetic map in cM comprised 12 LGs, 4849 markers, anchored 2381 scaffolds and 1285 CDS. Marker density is shown as intensity of gray within the LG bars, along with putative genes annotated in Polashock et al. (2014) displayed as colored squares (see Additional file 3: Table S5)

The average number of observed recombination events per gametic linkage was ~1 for both parental bin maps P_1_ and P_2_ (Table 2), which was expected for LGs with an average size of 100 cM. An average of 0.5 to 1 recombination events per chromosome were detected in *Rubus*, which is very similar to our findings [20]. Recombination ‘cold spots’ were observed mainly in LGs 8 and 11, especially in areas with few markers detected. Given that FISH experiments for cranberry are not available yet, the nature of the centromeres is not known (i.e. acrocentric versus metacentric chromosomes), therefore, cold spots could correspond to telomeric and/or centromeric regions (Fig. 5). We also observed an increased recombination rate in several maternal LGs (Additional file 4: Figure S2), and this translated into some of the LGs in the maternal (P_1_) linkage map being slightly longer than the LGs in the paternal map (P_2_; 20 cM shorter on average) due to the presence of higher recombination in the maternal bin map. Higher recombination rates in the maternal cranberry bin map could be due to genomic structural variation between genetic backgrounds such as has been documented in rapeseed [75] and maize [76]. Our study is one more example that recombination rates can be sex-dependent as reported in olive, apple, and grape [67, 77–79]. Interestingly, we found that length in cM and the number of recombination events were related to the extent of linkage disequilibrium in the parental maps (Table 2; Fig. 1).

### Linkage disequilibrium (LD)

In addition to the characterization of the recombination frequency between markers in the parental maps, this is the first study reporting linkage disequilibrium (LD) in a biparental population in cranberry. Information regarding linkage disequilibrium (LD) is widely available in model species with substantial genomic resources, but has yet to be described in most minor crops such as cranberry. A pseudo-testcross strategy recommended for outcrossing species was used to measure the LD as r^2^, the square correlation coefficient between two loci [80–83]. The LD patterns across all linkage groups were related to the calculated recombination frequencies. Linkage disequilibrium decaying across 50 cM was calculated. LD was broken down (considering *r*^2^ = 0.2) at ~32 cM in our cranberry population. Given the expected genome size and the fact that the markers covered 99.5 % of the genome, each cM in cranberry is equivalent to ~422 Kb, which means that the calculated cranberry extent of LD of 32 cM corresponds to ~13.39 Mb. This long-distance LD is typical for a biparental population where loci are in full linkage disequilibrium (Additional file 5: Figure S3; Table 2). Thus, as expected for a biparental population the LD decayed slowly. LD above 0.5 and 0.4 extended over 25 cM and 30 cM, respectively. The LD decay observed in our population is similar to biparental populations in other species [81]. However, it is known that other outcrossing species such as maize tend to have a short-distance LD when calculated using diversity panels or compendiums of RILs [81, 84]. Future in depth analyses of genome-wide cranberry LD will necessitate association and diversity panels to provide more information about LD decay in this perennial, outcrossing species. Such studies will reveal whether the different reproductive features of the species such as sexual reproduction by outcrossing, forced self-fertilization, and asexual propagation through stolons have played a role in the evolution of linkage disequilibrium in the species.

### Segregation distortion

Segregation distortion has been previously reported in cranberry by Georgi et al. [36] and Schlautman et al. [44] and by Rowland et al. [51] in blueberry, but this is the first time that segregation distortion was analyzed at genome-wide level using a fully saturated linkage map in the *Vaccinium* genus (Fig. 3). In our study, genome-wide segregation distortion was not always randomly distributed across the 12 LGs (Table 2; Fig. 3). Moreover, segregation distortion was observed in well-defined regions of the cranberry LGs, which in turn could have biological or evolutionary significance. For example, a preponderance of distorted markers were located in LGs 6, 7, 9,11 and 12 (19.7, 19.4, 12.7, 16.3 and 14.8 % of the markers, respectively) (Fig. 3). Conversely, linkage groups 2, 3, 4 and 5 possessed up to three to four times lower number of distorted markers than the rest of the LGs in the map, 4.8, 2, 5.4 and 4.3 % distorted markers, respectively (Fig. 3). Additionally, the high density of markers used in our study enabled us to accurately characterize regions of segregation distortion across the genomes of the parental (P_1_ and P_2_) bin maps. P_2_ map possessed over three times more distorted markers than the P_1_ map with markers positioned in LG6, LG7 and LG9 of the P_2_ map possessing the most elevated levels of distortion (Table 2). The distortion favored the homozygote configuration for the P_1_ (82 % of ABxAA distorted markers had more genotypes AA) and no specific allelic configuration was favored in P_2_ (56:44 % for AA:AB genotypes), but heterozygote configurations were more common in P_2_ (AAxAB) than in P_1_. Since P_2_ has an inbreeding coefficient of F = 0.125, whereas, P_1_ is estimated to be F = 0 this finding was according with observations. For example, in a 3 cM region of LG10 (cM 9-12) 17 distorted markers were clustered all favoring heterozygote genotypes over the homozygote configuration, but opposite situations favoring the homozygote configuration were observed as well. Segregation distortion in the parental and integrated maps may indicate lethal and sub-lethal genes that tend remain heterozygous as classical genetic studies have shown in raspberry [85]. Additionally, it has been reported that inversions, e.g., paracentric inversions, result in genetically unbalanced gametes that carry deletions, insertions, reducing fertility and leading to segreation distortion. Interestingly, LG9 (cM 13-16), LG11 (cM 1-6) and LG12 (cM 32-36) in our study showed strong patterns of segregation distortion in areas of putative inversions based on our collinearity analyses between cranberry parental maps [66]. As the genomic structure of cranberry is revealed, candidate genes in the distortion areas will allow the study of the inheritance of these chromosome regions revealing the forces that shaped the genome.

### Synteny analysis

We used a ‘Ben Lear’ draft nuclear genome [30] as reference for SNP calling in order to anchor cranberry scaffolds and construct pseudo-chromosomes for *Vaccinium* [86, 87]. The integrated linkage map allowed us to anchor 2381 scaffolds, about 1.08 % of the current number of scaffolds available, covering 13 Mb of the cranberry genome (~2.7 % of the total genome size), but representing 5 % of the CDS from the entire genome, and uniformly spread across the genome. This result reflects the high fragmentation of the current cranberry genome assembly and highlights the needs to improve the sequence contiguity. Nevertheless, the linkage map presented here and the anchored scaffolds represent a foundation for future efforts to build a high-quality cranberry genome assembly [30]. We used the scaffolds to position the putative genes annotated by Polashock et al. [30] in these pseudo-molecules (Fig. 5). Synteny analysis with other marker technologies such as EST-SSRs and AFLPs have been useful in other species using markers in the order of hundreds. With NGS technologies, initial synteny studies can be easily performed by using high density genetic maps, where thousands of genes can be positioned and compared to other related species [88, 89]. The syntenic comparisons between the cranberry map against kiwifruit, coffee, and grape genomes provided initial syntenic comparisons with these species (Fig. 4). By comparing the gene order of cranberry to kiwifruit, grape, and coffee, we found well-defined regions of synteny. Syntenic blocks were found between all cranberry LGs and the three other genomes, showing different levels of genetic relationship with the three species analyzed, and with kiwifruit being the most similar based on the number and size of the syntenic regions.

## Conclusion

The use of GBS methodologies allowed the identification of ~10,842 potential SNP’s, from which ~4849 were used to construct the first saturated linkage map in cranberry. Mapping methodologies and ordering algorithms for F_1_ crosses (pseudo-testcross strategy) were used to characterize the recombination frequency and build a linkage map. We created an integrated and parental bin maps (P_1_ x P_2_) and characterized linkage disequilibrium (LD) for the first time in cranberry. LD patterns were consistent with recombination frequencies in map and LD decayed at approximately 32 cM (*r*^2^ = 0.2), as expected for biparental populations. We estimated that each cranberry cM is equivalent to ~422 Kb. We provide all the sequences and marker positions that can be used for the *Vaccinium* community to perform fine mapping within a region of interest or as reference for comparative genomics with other species (Additional file 3: Table S5). Based on our current map, it was possible to anchor a total of 2381 scaffolds out of 229,000 comprising only ~13 Mb out of the 470 Mb estimated for the cranberry genome. Therefore, much deeper cranberry genome sequencing will be required in order to reduce the number of scaffolds to make the high density mapping strategy a feasible option for anchoring the genome into pseudo-molecules representing cranberry chromosomes. Putative genes annotated during the previous genome sequencing efforts were positioned into the map and these genes were used to perform an initial comparative synteny analysis of cranberry with kiwifruit, grape and coffee providing a first insight into the cranberry homology with related species. In summary, we used GBS to rapidly and reliably generate substantial genomic information, which will serve as a starting point for QTL mapping studies in this cranberry mapping population.

## Methods

### Plant material and DNA extraction

Genetic analyses were performed using a full-sib segregating population of 362 cranberry progeny from a cross of two elite cranberry selections, *[BGx(BLxNL)]95* (P_1_) and *GH1x35* (P_2_), selected due to their phenotypic differences for agronomic traits of interest. Both parental and progeny clones are maintained by the Valley Corporation in Tomah, WI. Total genomic DNA from 0.1 g of leaf tissue was extracted from fresh leaves of single uprights for each of the accessions using a modified CTAB protocol [90].

### Genotyping by sequencing

Experion® (BioRad, Hercules, CA) traces showing fragment distribution sizes of libraries made with ApeKI (4-base cutter), EcoT22I (6-base cutter), and PstI (6-base cutter), commonly used to reduce genome complexity according to Elshire et al. [1], were compared to optimize the GBS pipeline in cranberry. Experion® traces confirmed that the majority of the digested fragments were within ~160-500 bp (fragments > ~500 bp will not be sequenced on the Illumina platform) for all three libraries (Additional file 7: Figure S5). A peak suggesting highly repetitive DNAs was observed in the PstI library; however, these fragments represented a small portion of the library. There was no evidence of highly repetitive DNAs (strong peaks) in the ApeKI or EcoT22I libraries.

ApeKI cut the genome most frequently and the highest amount of SNPs would probably be identified in libraries made with this library. Nevertheless, sequence coverage per SNP locus, will likely be lower compared to libraries prepared with the 4-base cutter enzymes. Past studies in other crops suggest that fewer overall SNP calls will be made with the EcoT22I library compared to the PstI library; however, tighter fragment size distribution observed in the Experion® traces of the EcoT22I library suggested that sequence coverage would likely be higher for EcoT22I.

EcoT22I, which cuts the site 5′-ATGCA↓T-3′//3′-T↑ACGTA-5′, was selected for reducing genome complexity in this study based on optimization results in cranberry (Additional file 7: Figure S5) to ensure appropriate coverage for sequence tags in the 364 pseudo-testcross progeny and their parents. Unique barcodes, from 5 to 10 bp long, were ligated to fragments to differentiate reads generated for each cranberry genotype according to Elshire et al. [1] protocol. Adapters were designed for either single or pair-end Illumina sequencing which did not contain the EcoT221 recognition site and that would not regenerate EcoT221 recognition sites after ligation to genomic DNA. Resulting libraries were sequenced on the Illumina HiSeq 2000 sequencing platform (Illumina, San Diego, California). SNPs generated in this study were submitted to the National Center of Biotechnology Information (NCBI) and the NCBI_ss# can be found in the Additional file 8.

To determine whether sequence coverage was related to sample position in the 96-well plates during library preparation or not, a linear model of the form y = Xβ + ε was fitted, where X is the design matrix for the fixed effects such as plates, rows nested in plates and columns nested in plates, β is the vector of fixed effects corresponding to those mentioned previously and ε is the vector of random errors associated to the measurements. We checked for any violation to the Gauss-Markov assumptions such as linearity, and multivariate normality distribution, homogeneity of variance, multicollinearity, and random distribution of the errors.

### Marker filtering, imputation, and distortion

The TASSEL GBS Bioinformatics pipeline was used to filter raw sequences, align and merge sequence tags by genotype, and call SNPs in the resulting data. SNP calls were made using *de novo* methods in aligned sequence tags and by mapping tags to their physical location in a nuclear genome assembly [30]. The resulting data was converted to HapMap format for use in further genetic analyses.

SSR primer pairs previously developed by Polaschock and Vorsa [91], Boches et al. [92], Georgi et al. [93], Zhu et al. [94] and Schlautman et al. [37] and positioned in the Schlautman et al. [44] cranberry linkage map were also used to genotype the 362 cranberry progeny in this study (Additional file 3: Table S5, Table 1). Multiplex PCR reactions, fragment analysis, and allele scoring were performed according to Schlautman et al. [44].

R scripts were used to remove SNP and SSR markers with excessive missing data (>20 %) to avoid the loss of accuracy during the imputation process [13], and markers with a minor allele frequency (MAF) < 0.10 were removed as well. To maximize the amount of SNPs and genotypic data available for linkage mapping, simulations using median, principal components and linear discriminant imputation methods were tested. Linear discrimination analysis (LDA) imputation yielded the lowest classification error (9.65 %) of the methods tested after performing extensive simulations. Therefore, linear discriminant imputation was performed using the first 80 Linear Discriminants (LDs) to impute missing marker values in this study. Following imputation, extremely distorted loci were identified using Chi-square tests (*p*-value < 0.001), and such markers were removed from the study.

### Recombination estimation and high density linkage mapping

The imputed marker data was separated into 2 unique sets of uniparental configurations (ABxAA and AAxAB) to create starting parental maps for P_1_ and P_2_ and initially ran in JoinMap 4.1® by removing all identical loci. Data was extracted and phased to be used as a DH population type and transformed to ASMap format for DH populations [42]. The MST algorithm available in ASMap was used to detect genotyping error and to create bins of markers for parental maps. Marker ordering obtained for the parental bin maps was used to force the inclusion of double heterozygous markers (ABxAB) and develop an integrated map using JoinMap 4.1®. Linkage groups were determined with a LOD threshold > 10, recombination fraction threshold of 0.35, ripple value of 1.0, jump threshold of 3.0, and a triplet threshold of 5.0; and Kosambi’s mapping function was used to calculate genetic distances among loci. Marker order and presence of high number of false double recombination events in the parental bin maps was further interrogated using the *colorize* option to view graphical genotypes and the *genotyping probabilities* tab sheet in JoinMap 4.1®, markers causing discrepancies were manually removed.

Recombination matrices were calculated and plotted to validate the most parsimonious marker order found by the MST algorithm. Graphical presentations of parental maps were prepared using MapChart v2.2 to inspect collinearity between the current integrated map and the previous SSR map developed from Schlautman et al. [44].

Genome length (*G*_*O*_) was calculated by adding individual lengths of all linkage groups. The expected length of each linkage group was estimated according to method four of Chakravarti et al. [70] by inflating the observed map length (cM) by a factor of (*m* + 1)/(*m*-1) where *m* is the number of mapped makers in the linkage group. The expected genome length *(G*_*E*_*)* was then estimated summing the estimated linkage group lengths. Observed genome coverage *(GC*_*O*_*)* was calculated as the ratio of *G*_*O*_ and *G*_*E*_ [95]_._

### Linkage disequilibrium (LD) and LD decay

Linkage disequilibrium in the cranberry genome was analyzed using the two uniparental datasets generated for parental linkage bin mapping. The markers were sorted according to their positions in the parental linkage maps (recombination based). Linkage disequilibrium (r^2^) [81], defined as the square of the correlation coefficient between the two loci was calculated as ${r}^2=\frac{{\left({\mathrm{D}}_{\mathrm{ab}}\right)}^2}{\uppi_A{\uppi}_a{\uppi}_B{\uppi}_b}$ where, considering a pair of loci with alleles *A* and *a* at locus one and *B* and *b* at locus two with allele frequencies π_A_, π_a_, π_B_, and π_b_ respectively and haplotype frequencies π_*AB*_, π_*Ab*_, π_*aB*_, and π_*ab*_, the difference between the observed and expected haplotype frequencies is *D* = π_*AB*_ − π_*A*_π_*B*_. LD plots were obtained to visually assess the extent of r^2^ within the cranberry linkage groups and genome, and to determine if the calculated LD correlated with the marker positions in the high density linkage map. To assess the genome-wide rate of LD decay, marker positional information from all 12 linkage groups was combined into a single data frame, and r^2^ was computed for bins of 5 cM across to assess LD decay for each parental bin map.

### Analysis of genome-wide segregation distortion

Chi-square analyses of segregation distortion implemented in JoinMap 4.1® [43] were performed for all loci. Loci were plotted according to their positions in the integrated linkage map and chi-square statistic from the segregation distortion test, and the loci were colored based on the significance of their chi-square test statistic in order to visually genome-wide segregation distortion. Regions of distorted loci were identified and reported.

### Scaffold anchoring

Using the position information generated by the high density map we order the scaffolds containing such markers, and such sequences were merged to create pseudo-molecules. Using R capabilities, we generated additional files containing the marker, position in cM, scaffold origin, sequence, and genes annotated for such scaffold to be used in the synteny analysis (Additional file 3: Table S5).

### Synteny analysis

A local BLASTN search of anchored cranberry CDS within the grape (NCBI ID 401), coffee [95], kiwifruit (NCBI ID 16401) and blueberry [29] genomes was performed to identify regions of conserved synteny between the genomes. A minimum expectation value of 10e^-10^, an alignment score greater than 80, and a minimum alignment length of 50 bp were used as parameters for declaring real BLAST hits. When a pair of cranberry CDS were located less than 10 cM apart on a linkage group and their putative homologous sequences were within 10 Mbp in the genome of the reference species, the regions were considered to be putative regions of conserved microsynteny. However, additional putative regions of macrosynteny spanning large distances were manually identified by visually plotting the BLAST hits in the cranberry LGs vs. the reference species genomes in CIRCOS [96].

## Abbreviations

CDS, coding DNA sequence; cM, centiMorgan; CP, cross pollination; DH, double haploid; F_1_ cross, cross of two highly heterozygous parents; GBS, genotyping by sequencing; LDA, linear discriminant analysis; LG, linkage group; LOD, Logarithm (base 10) of odds; MST, minimum spanning tree; PCR, Polymerase chain reaction; QTL, quantitative trait loci; SNPs, single nucleotide polymorphisms; SSRs, simple sequence repeats; *V. macrocarpon*, *Vaccinium macrocarpon* Ait.

## Additional files

Additional file 1: ANOVA tables for testing the influence of plate, row, column and sample in the percentage (%) of missing data in the experiment. (DOCX 172 kb) Additional file 2: Figure S1. Parental bin maps. In red the bin map for *[BGx(BLxNL)]95* is shown, and the bin map for *GH1x35* in blue. (PDF 396 kb) Additional file 3: Additional summary tables (cited in manuscript text) for the chracterization of genomic features through hight density linkage mapping. (XLSX 983 kb) Additional file 4: Figure S2. Integrated and parental maps homology. Homology between the integrated map (blue) and the parental maps (P_1_ in red, P_2_ in green) is shown. Homology between markers is indicated with black solid lines. (JPG 241 kb) Additional file 5: Figure S3. LD decay. Linkage disequilibrium decay as a function of distance in cM is presented for each parental map (P_1_ in blue, P_2_ in orange). Linkage disequilibrium (measured as r^2^) decreases as a function of physical distance (in cM). In the biparental population, loci are in full linkage disequilibrium causing a slow decay. (JPEG 265 kb) Additional file 6: Figure S4. Synteny analysis between cranberry and grape. CIRCOS plots highlights syntenic regions and genomics rearrangements between cranberry and grape using CDS information anchored by the SSR-SNP map. (JPG 6129 kb) Additional file 7: Figure S5. Experion® traces for three restriction enzymes. Traces for restriction enzymes APKI, EcoT22I and PstI displayed show the fragment size obtained by digesting cranberry DNA with a particular enzyme. Fragments below 500 bp are ideal for the GBS pipeline. (JPG 137 kb) Additional file 8: Generated SNPs submitted to the National Center of Biotechnology Information (NCBI) and the NCBI_ss#. (TXT 322 kb)
