# Supplementary material for: Exploiting genotyping by sequencing to characterize the genomic structure of the American cranberry through high-density linkage mapping
Source: BMC Genomics. 2016 Jun 13;17:451. doi: 10.1186/s12864-016-2802-3 (PMC4906896; doi:10.1186/s12864-016-2802-3)
Supplement: Additional file 1: — ANOVA tables for testing the influence of plate, row, column and sample in the percentage (%) of missing data in the experiment. (DOCX 172 kb) [file 12864_2016_2802_MOESM1_ESM.docx]

Additional File 1

ANOVA tables for testing the influence of plate, row, column and sample in the percentage (%) of missing data in the experiment.

Analysis of Random effects Table for testing σ^2^_p_ > 0 (plate variation is greater than zero) using likelihood ratio test (LRT) from the model y_ij_= µ + p_i_ + ε_ij_, where µ is the intercept, p_i_ is the plate effect and ε_ij_ is the random error associated to the measure.

|  | Variance comp. | Chi.sq under Ho | Chi.DF | p.value |
| --- | --- | --- | --- | --- |
| Plate | 12.93 | 323 | 1 | <2e-16 *** |
| --- |  |  |  |  |


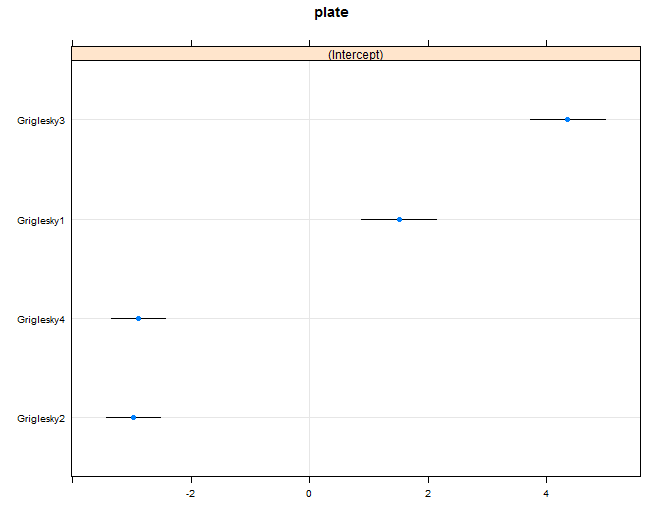


Analysis of Random effects Table for testing σ^2^_r_ > 0 (row in plate variation is greater than zero) using likelihood ratio test (LRT) from the model y_ij_= µ + r_i_ + ε_ij_, where µ is the intercept, r_i_ is the row effect and ε_ij_ is the random error associated to the measure.

|  | Variance comp. | Chi.sq under Ho | Chi.DF | p.value |
| --- | --- | --- | --- | --- |
| Row | 0.6947 | 9.67 | 1 | 0.002 ** |
| --- |  |  |  |  |


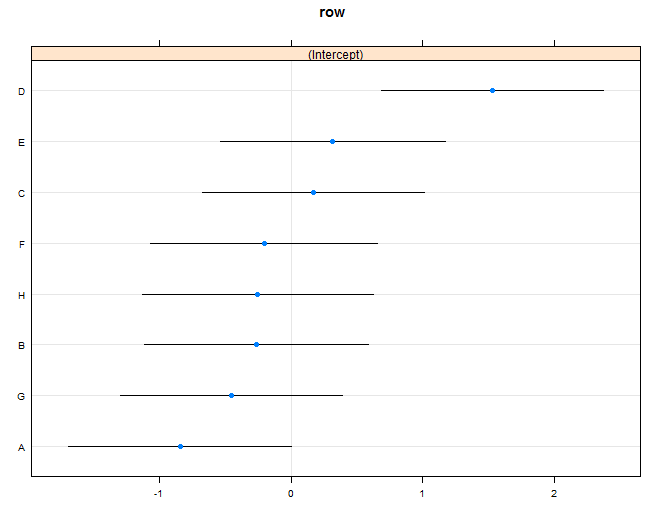


Analysis of Random effects Table for testing σ^2^_c_ > 0 (column in plate variation is greater than zero) using likelihood ratio test (LRT) from the model y_ij_= µ + c_i_ + ε_ij_, where µ is the intercept, c_i_ is the column effect and ε_ij_ is the random error associated to the measure.

|  | Variance comp. | Chi.sq under Ho | Chi.DF | p.value |
| --- | --- | --- | --- | --- |
| Column | 2.003 | 36.6 | 1 | 1e-09 *** |
| --- |  |  |  |  |


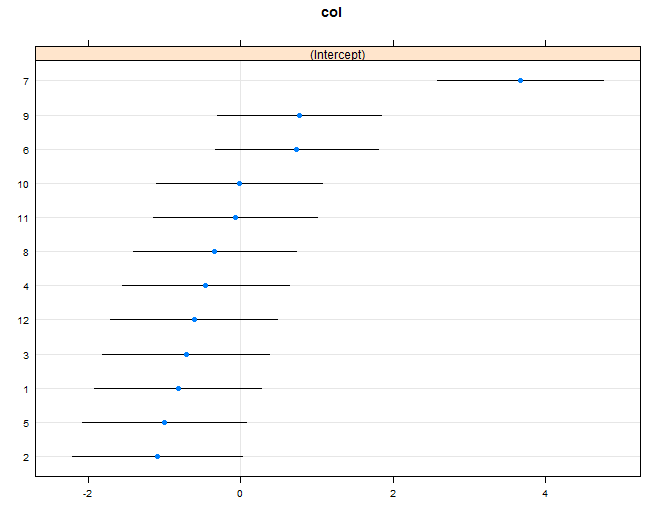


Analysis of Random effects Table for testing σ^2^_g_ > 0 (genotype variation is greater than zero for missing data) using likelihood ratio test (LRT) from the model y_ij_= µ + g_i_ + ε_ij_, where µ is the intercept, g_i_ is the genotype or sample effect and ε_ij_ is the random error associated to the measure.

|  | Variance comp. | Chi.sq under Ho | Chi.DF | p.value |
| --- | --- | --- | --- | --- |
| Plant sample | 16.21 | 5872 | 1 | <2e-16 *** |
| --- |  |  |  |  |


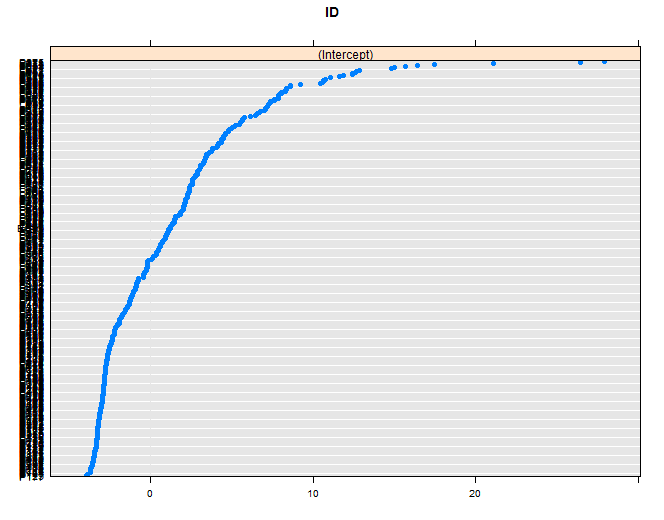


ANOVA tables for testing the influence of plate, row, column and sample in the number of reads per sample in the experiment.

Analysis of Random effects Table for testing σ^2^_p_ > 0 (plate variation is greater than zero) using likelihood ratio test (LRT) from the model y_ij_= µ + p_i_ + ε_ij_, where µ is the intercept, p_i_ is the plate effect and ε_ij_ is the random error associated to the measure.

|  | Variance comp. | Chi.sq under Ho | Chi.DF | p.value |
| --- | --- | --- | --- | --- |
| Plate | 7.579e+10 | 29.5 | 1 | 6e-08 *** |
| --- |  |  |  |  |


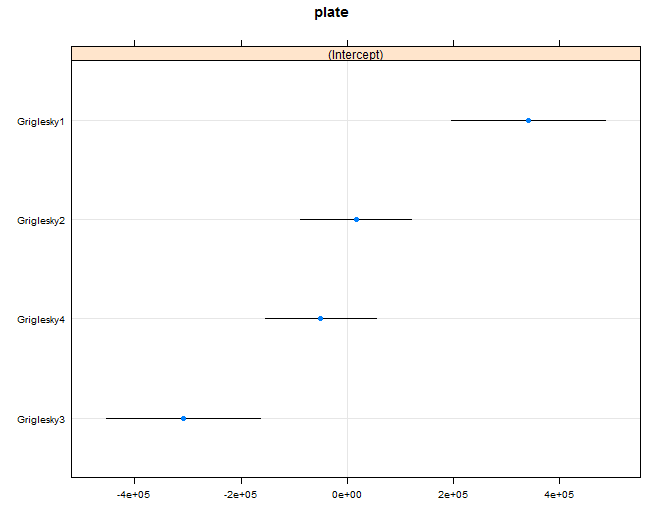


Analysis of Random effects Table for testing σ^2^_r_ > 0 (row in plate variation is greater than zero) using likelihood ratio test (LRT) from the model y_ij_= µ + r_i_ + ε_ij_, where µ is the intercept, r_i_ is the row effect and ε_ij_ is the random error associated to the measure.

|  | Variance comp. | Chi.sq under Ho | Chi.DF | p.value |
| --- | --- | --- | --- | --- |
| Row | 2.197e+10 | 9.41 | 1 | 0.002 ** |
| --- |  |  |  |  |


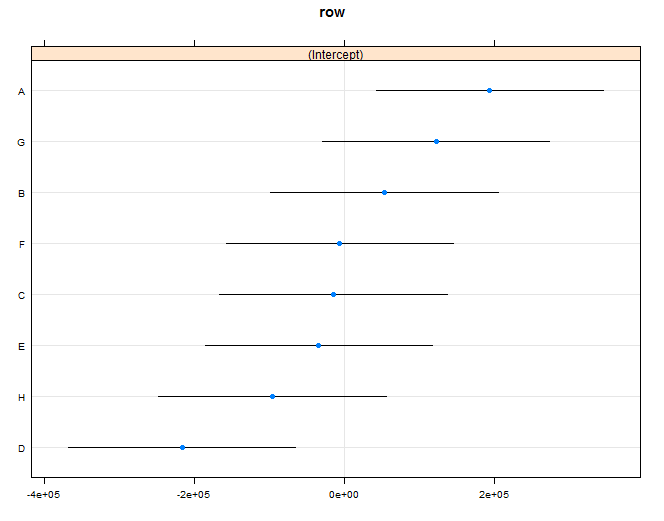


Analysis of Random effects Table for testing σ^2^_c_ > 0 (column in plate variation is greater than zero) using likelihood ratio test (LRT) from the model y_ij_= µ + c_i_ + ε_ij_, where µ is the intercept, c_i_ is the column effect and ε_ij_ is the random error associated to the measure.

|  | Variance comp. | Chi.sq under Ho | Chi.DF | p.value |
| --- | --- | --- | --- | --- |
| Column | 6.344e+10 | 37.4 | 1 | 1e-09 *** |
| --- |  |  |  |  |


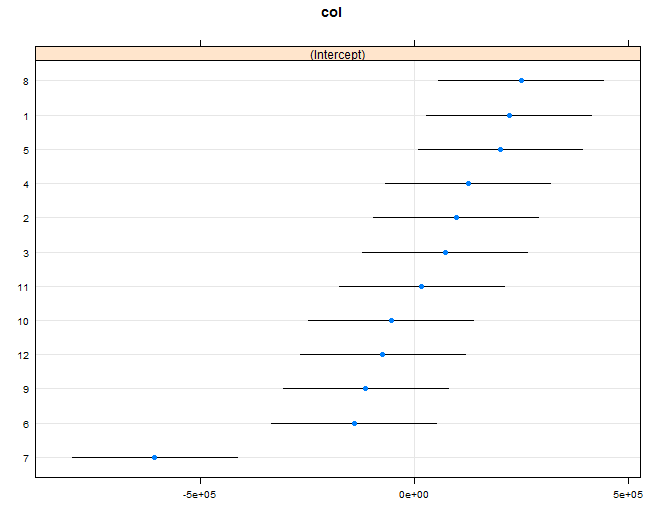


Analysis of Random effects Table for testing σ^2^_g_ > 0 (genotype variation is greater than zero for missing data) using likelihood ratio test (LRT) from the model y_ij_= µ + g_i_ + ε_ij_, where µ is the intercept, g_i_ is the genotype or sample effect and ε_ij_ is the random error associated to the measure.

|  | Variance comp. | Chi.sq under Ho | Chi.DF | p.value |
| --- | --- | --- | --- | --- |
| Plant sample | 1.152e+10 | 19.3 | 1 | 1e-05 *** |
| --- |  |  |  |  |


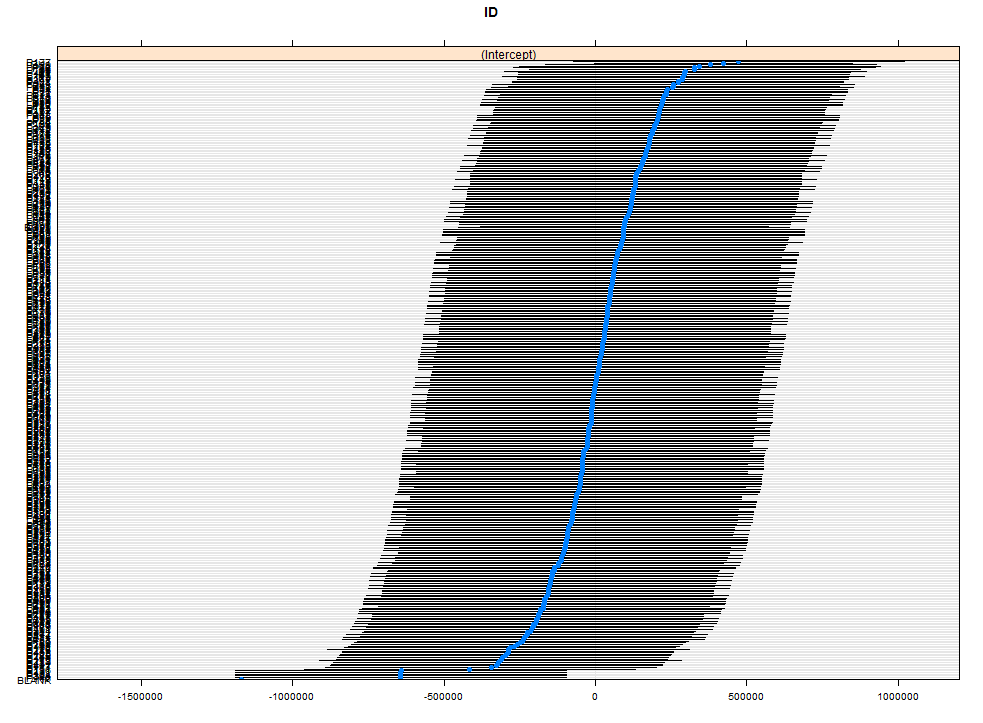


| **Tab for total reads**. Below the total numer of reads is displayed. | |
| --- | --- |
|  |  |
|  |  |
|  | C50VDACXX_5_fastq.gz |
| Number of samples | 96 |
| Number reads in lane | 288333754 |
| Number reads in lane | 225413371 |
| Number reads in lane | 24697707 |
|  | C50VDACXX_6_fastq.gz |
| Number of samples | 96 |
| Number reads in lane | 298516524 |
| Number reads in lane | 145611175 |
| Number reads in lane | 22761121 |
|  | C50VDACXX_7_fastq.gz |
| Number of samples | 96 |
| Number reads in lane | 295267589 |
| Number reads in lane | 158106631 |
| Number reads in lane | 23593690 |
|  | C50VDACXX_8_fastq.gz |
| Number of samples | 96 |
| Number reads in lane | 294163263 |
| Number reads in lane | 132178581 |
| Number reads in lane | 23476794 |
|  | C511KACXX_2_fastq.gz |
| Number of samples | 96 |
| Number reads in lane | 269645508 |
| Number reads in lane | 237822045 |
| Number reads in lane | 20279965 |
|  | C511KACXX_3_fastq.gz |
| Number of samples | 96 |
| Number reads in lane | 278757339 |
| Number reads in lane | 237991986 |
| Number reads in lane | 24225523 |
|  |  |
|  | total number of reads |
|  | 3000842566 |
|  |  |

| **Tab_reads. Total number of reads per plate sent for GBS for the parent of the biparental population.** Each parent was included twice in each plate as a control measure and to guarantee the correct coding in the F1 cross. | | | | | | | | | | | | | | | |
| --- | --- | --- | --- | --- | --- | --- | --- | --- | --- | --- | --- | --- | --- | --- | --- |
|  |  |  |  | |  | |  |  |  |  |  |  |  |  |  |
|  | **GH1x35** |  | | **[BGx(BLxNL)]95** | | |  |  |  |  |  |  |  |  |  |
| PLATE1 | 3067144 | reads | | 2444095 | | reads |  |  |  |  |  |  |  |  |  |
| PLATE1 | 2492710 | reads | | 1584788 | | reads |  |  |  | **Total number of reads:** | 3E+09 |  |  | 384 | siblings |
| PLATE2 | 2124454 | reads | | 1994828 | | reads |  |  |  | **Number of reads for both parents:** | 40563518 |  |  | 10 | failed samples |
| PLATE2 | 1579975 | reads | | 1226994 | | reads |  |  |  | **Total reads for all siblings:** | 2.96E+09 |  |  | 12 | parentsamples |
| PLATE3 | 3926885 | reads | | 2683988 | | reads |  |  |  |  |  |  |  | 4 | blanks, one per plate |
| PLATE3 | 2993944 | reads | | 2254510 | | reads |  |  |  |  |  |  |  | 358 | utilized |
| total | 16185112 |  | | 12189203 | | = | 28374315 |  |  | **Average number of reads per individual** | 8268936 |  |  |  |  |
