# Supplementary figures and images for: Exploiting genotyping by sequencing to characterize the genomic structure of the American cranberry through high-density linkage mapping
Source: BMC Genomics. 2016 Jun 13;17:451. doi: 10.1186/s12864-016-2802-3 (PMC4906896; doi:10.1186/s12864-016-2802-3)

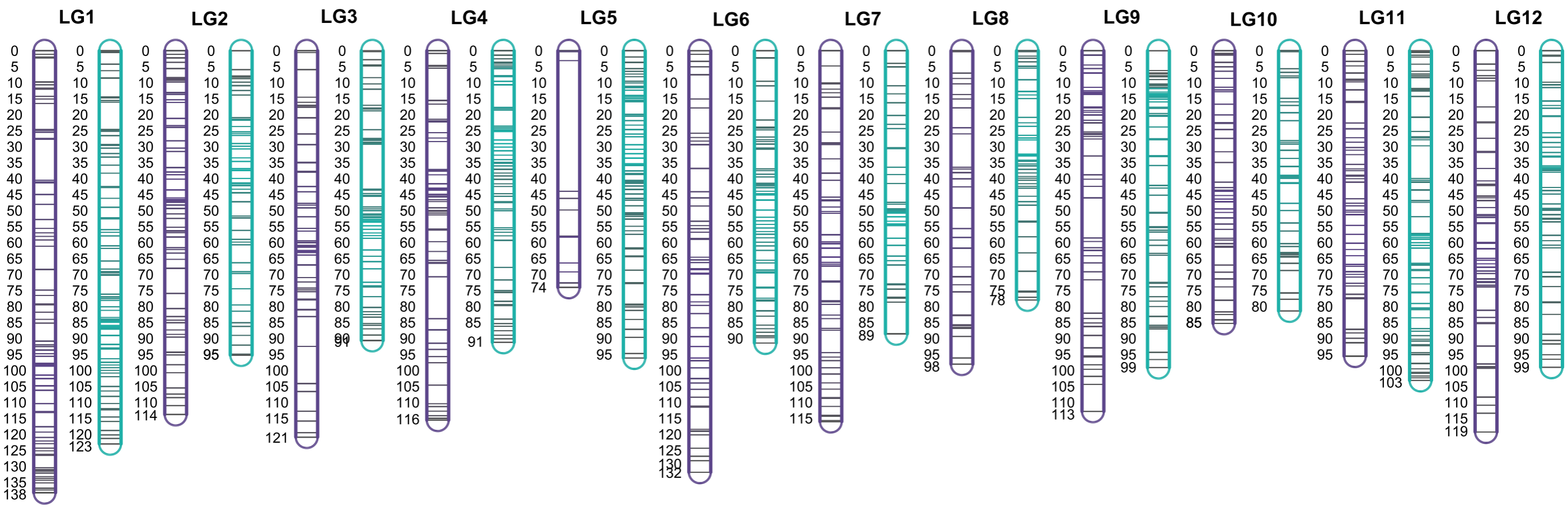

Supplement: Additional file 2: Figure S1. — Parental bin maps. In red the bin map for [BGx(BLxNL)]95 is shown, and the bin map for GH1x35 in blue. (PDF 396 kb) [file 12864_2016_2802_MOESM2_ESM.pdf]

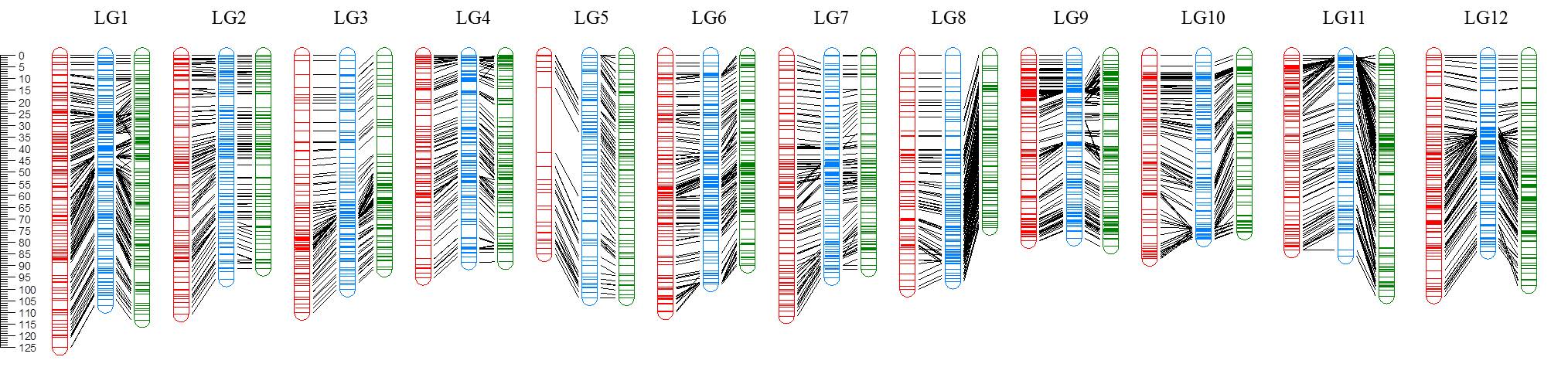

Supplement: Additional file 4: Figure S2. — Integrated and parental maps homology. Homology between the integrated map (blue) and the parental maps (P1 in red, P2 in green) is shown. Homology between markers is indicated with black solid lines. (JPG 241 kb) [file 12864_2016_2802_MOESM4_ESM.jpg]

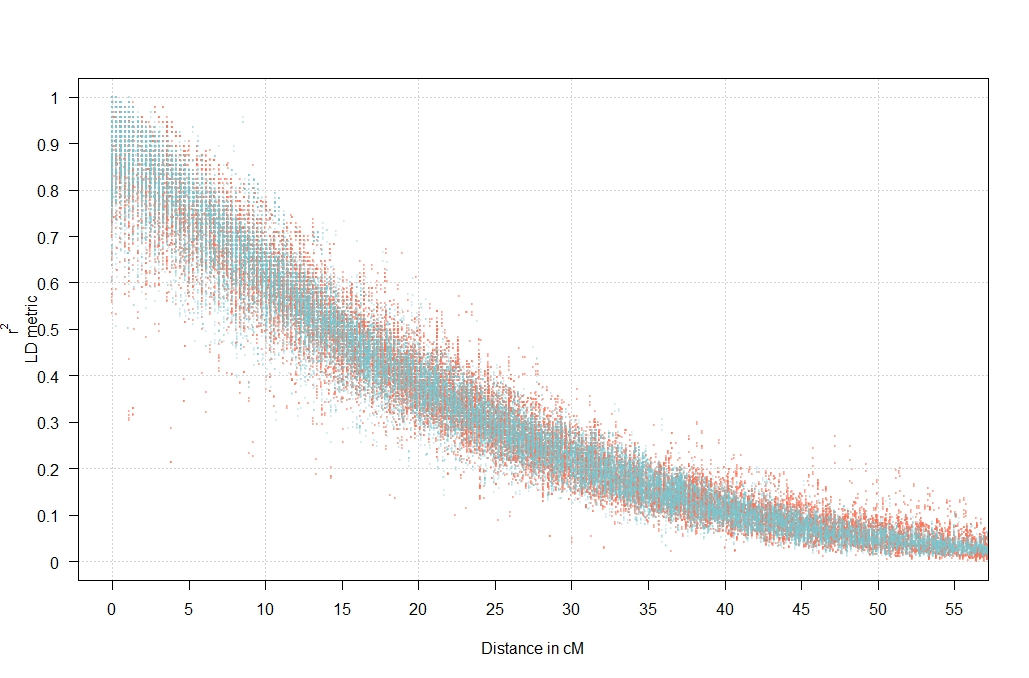

Supplement: Additional file 5: Figure S3. — LD decay. Linkage disequilibrium decay as a function of distance in cM is presented for each parental map (P1 in blue, P2 in orange). Linkage disequilibrium (measured as r2) decreases as a function of physical distance (in cM). In the biparental population, loci are in full linkage disequilibrium causing a slow decay. (JPEG 265 kb) [file 12864_2016_2802_MOESM5_ESM.jpeg]

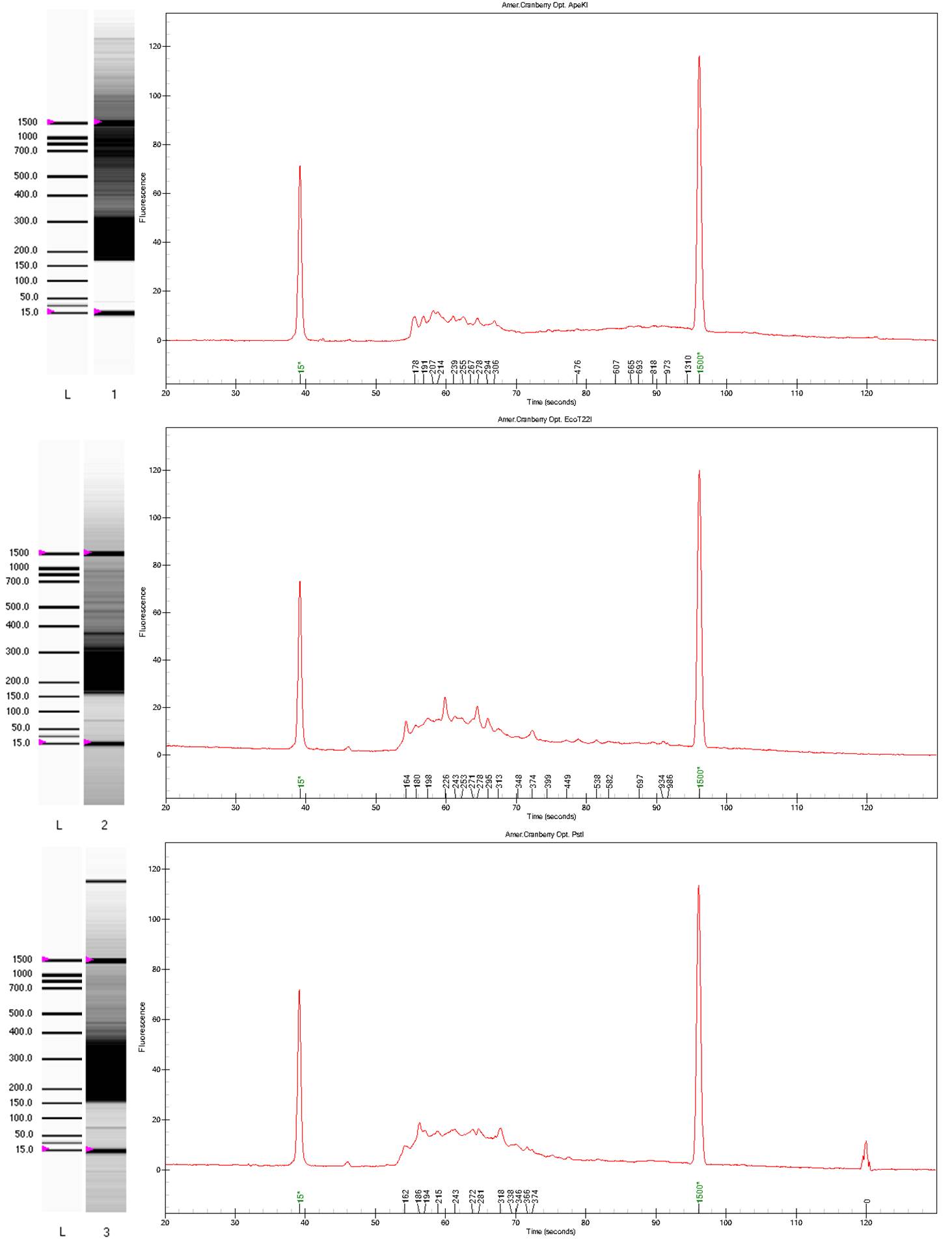

Supplement: Additional file 7: Figure S5. — Experion® traces for three restriction enzymes. Traces for restriction enzymes APKI, EcoT22I and PstI displayed show the fragment size obtained by digesting cranberry DNA with a particular enzyme. Fragments below 500 bp are ideal for the GBS pipeline. (JPG 137 kb) [file 12864_2016_2802_MOESM7_ESM.jpg]
